# The Risk of Premature Burial

**Published:** 1914-06-13

**Authors:** 


*To the Editor of* The Hospital.

### The Risk of Premature Burial.

*To the Editor of* The Hospital.

Sir,—With reference to the letter of "Surgeon" in your issue of the 30th ult., may I venture to remark that since, in the great majority of cases, medical men do not employ scientific tests, artificial respiration, or any other means for resuscitation before certifying a person's death, how can they avoid making mistakes, as they not infrequently do, and pronounce the living to be dead? That the risks of premature burial are real and worthy of the greatest consideration is proved by the careful personal investigations of the most trustworthy authorities, including Winslow, Bruhier, Anthony Fothergill, Hufeland, Struve, Marcus Herz, Köppen, Kite, Curry, Bouchût, Londe, Lénormand, Gannal, Gaubert, Moore Russell Fletcher, Alex. Wilder, Madden, Franz Hartmann, Icard, and many others, to say nothing of the facts reported from time to time to the Association for the Prevention of Premature Burial. Over 9,000 bodies are annually buried in England and Wales, and a much larger proportion in Scotland and Ireland, without a medical certificate of any sort, and it is impossible to know whether some were not consigned to the grave apparently, but not really, dead.

The fact that, notwithstanding the very stringent regulations in Germany for ascertaining that death has occurred before the removal of a body to the waiting mortuary, authorities like M. Gaubert and the Mayor of Munich have collected cases of revivification in these useful establishments, is striking proof that in some instances it is impossible to diagnose whether a person is dead or only apparently so until either resuscitation or putrefactive decomposition takes place. In countries like England, where there is no safeguard whatever against premature burial, the dangers of premature burial are ever present, and call for the immediate attention of Parliament. Those who desire to know the true facts concerning this urgent reform should study carefully the second edition of "Premature Burial and how it may be Prevented," by Walter R. Hadwen, M.D., etc., and the extensive literature of the aforesaid Association.—I am, Sir, yours respectfully,

June 2, 1914. Burial Reformer.

[This correspondence must now close.—Ed. The Hospital.]

